# Phytotracker, an information management system for easy recording and tracking of plants, seeds and plasmids

**DOI:** 10.1186/1746-4811-8-43

**Published:** 2012-10-13

**Authors:** Jeroen Nieuwland, Emily Sornay, Angela Marchbank, Barend HJ de Graaf, James AH Murray

**Affiliations:** 1 Cardiff School of Biosciences, Cardiff University, Museum Avenue, Cardiff, CF10 3AX, United Kingdom

**Keywords:** Database, Plant, LIMS, Software

## Abstract

**Background:**

A large number of different plant lines are produced and maintained in a typical plant research laboratory, both as seed stocks and in active growth. These collections need careful and consistent management to track and maintain them properly, and this is a particularly pressing issue in laboratories undertaking research involving genetic manipulation due to regulatory requirements. Researchers and PIs need to access these data and collections, and therefore an easy-to-use plant-oriented laboratory information management system that implements, maintains and displays the information in a simple and visual format would be of great help in both the daily work in the lab and in ensuring regulatory compliance.

**Results:**

Here, we introduce ‘Phytotracker’, a laboratory management system designed specifically to organise and track plasmids, seeds and growing plants that can be used in mixed platform environments. Phytotracker is designed with simplicity of user operation and ease of installation and management as the major factor, whilst providing tracking tools that cover the full range of activities in molecular genetics labs. It utilises the cross-platform Filemaker relational database, which allows it to be run as a stand-alone or as a server-based networked solution available across all workstations in a lab that can be internet accessible if desired. It can also be readily modified or customised further. Phytotracker provides cataloguing and search functions for plasmids, seed batches, seed stocks and plants growing in pots or trays, and allows tracking of each plant from seed sowing, through harvest to the new seed batch and can print appropriate labels at each stage. The system enters seed information as it is transferred from the previous harvest data, and allows both selfing and hybridization (crossing) to be defined and tracked. Transgenic lines can be linked to their plasmid DNA source. This ease of use and flexibility helps users to reduce their time needed to organise their plants, seeds and plasmids and to maintain laboratory continuity involving multiple workers.

**Conclusion:**

We have developed and used Phytotracker for over five years and have found it has been an intuitive, powerful and flexible research tool in organising our plasmid, seed and plant collections requiring minimal maintenance and training for users. It has been developed in an Arabidopsis molecular genetics environment, but can be readily adapted for almost any plant laboratory research.

## Background

Growing plants in a laboratory environment creates several logistical and administrative challenges. A system is needed to record and track the various plants and seed stocks from different researchers over time. Seeds can be stored for extended periods and laboratories will accumulate a large number of seed stocks which require careful and consistent documentation. Furthermore, national regulations require research groups to maintain records of genetically modified seeds and plants.

Laboratory Information Management Systems (LIMS) are designed to support a modern laboratory and often include a range of integrated features [1]. Some of these have been specifically aimed to store information about plasmids, microarrays, and even plants [2-5]. Furthermore we found LIMS which focus on plant breeding and field trials [6,7] and we know that some labs work with systems that are not publicly available. The previously reported solution PlantDB [2] offers a broadly similar solution, but being built in Microsoft Access, it is not cross-platform, less readily adaptable, and differs somewhat in the workflow conception. It also lacks the ability to link to details of plasmids encoding transforming DNA. Therefore, after evaluation of the existing options available to us, we could not find a simple, inexpensive yet adaptable system that can organise and track the operations involved in growing plants in a research facility. The system described here called ‘Phytotracker’ is designed to be easy to use and can be readily implemented in any laboratory without specialist computer knowledge. Phytotracker has been designed specifically for the end user and has been refined using regular feedback over a five-year period in a modern plant research laboratory.

### Implementation

The Phytotracker package has been developed in the commercially available database client FileMaker Pro. SQL-type web-based and free database systems such as MySQL were considered but FileMaker Pro was chosen because for the non-expert it is easier and faster to build a relatively small system in FileMaker Pro than in SQL-based systems. As a commercial package, Filemaker Pro is continually updated and supported and has extensive documentation ( http://www.filemaker.com). Although there are costs associated with FileMaker Pro, discounts normally apply to academic users, and we felt it allows for an intuitive and flexible interface that is highly scalable as systems grow and easily modified by non-experts. FileMaker Pro can be installed on both PC and Mac computers or on a server and works well in a mixed PC/Mac environment, and offers modification of fields and other features together with inherent FileMaker features such as report generation and searching. To facilitate simple labelling of seed bags and plants, cost effective DYMO label printers were used, but the label layout can be adapted for any printer. Phytotracker consists of 5 related tables containing the information of plants, trays, seeds, seed stocks and plasmids. Further technical details about the tables can be found in additional file 1.

Phytotracker, like any FileMaker database can be run on a server, shared or run as a stand-alone application. In our group we are running Phytotracker on a FileMaker Server, which allows the system to be available throughout the local network for any computer with FileMaker Pro installed. We have chosen the networked solution because researchers can access Phytotracker from their desks, and having dedicated computers with label printers in the growth rooms and potting area greatly facilitates harvesting and sowing plants, as labels can be printed on site.

## Results and discussion

### Database structure

**Figure 1:**
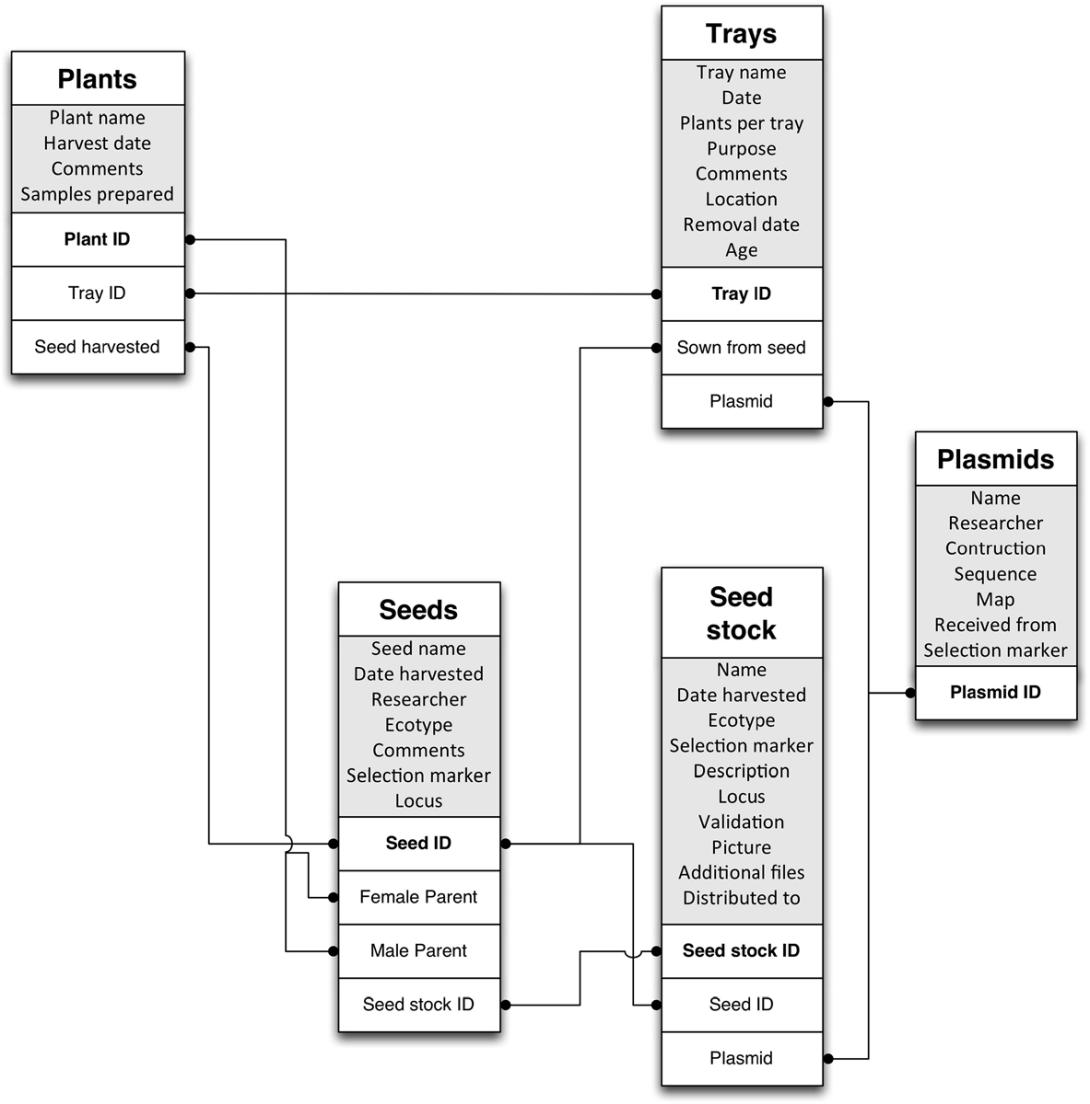
** Simplified conceptual Phytotracker database structure.** Grey boxes show a selection of important fields within the table. White boxes show key fields that are used for the relationships between the tables.

Phytotracker has been designed primarily around two stages of plant life: seeds and plants. Each table has at least one relationship with the seeds and/or plants tables (Figure 1). Phytotracker is a relational database and uses these multiple relationships to link information from five different tables. For example a record in the *Plants* table can have two relationships with the *Seeds* table, one identifying from which seed batch the plant has been grown and one identifying the seed batch that has been harvested from this plant. This allows easy tracking of the seeds used for growing a plant and which seeds have been harvested from it.

In principle, information can be entered in any table, however initial input into the database is usually via the *Seeds* table. When seeds are sown and the information is entered into the *Seeds* table, content is passed on to the *Plants* table which contains details of all plants that are currently growing or were grown in the past. Each plant record in this table has a unique ID number and refers to an individual plant in the growth room or glasshouse. When seeds are harvested from these plants, Phytotracker automatically generates a new unique seed record passing on the information from the parent plant and indicates the plant as harvested and removed from the growth facility. In this way, users can backtrack their plant lines and keep track of their plants growing at the moment. The *Trays* table can be simply seen as a collection of plants that are grown together, usually physically grouped in a pot(s) or a multi-compartment tray. This makes it simpler and quicker to enter and track information about similar plants grown at the same time, and is flexible in the number of plants in a “tray”, which can be set to 1 or any other value.

### General workflow

**Figure 2:**
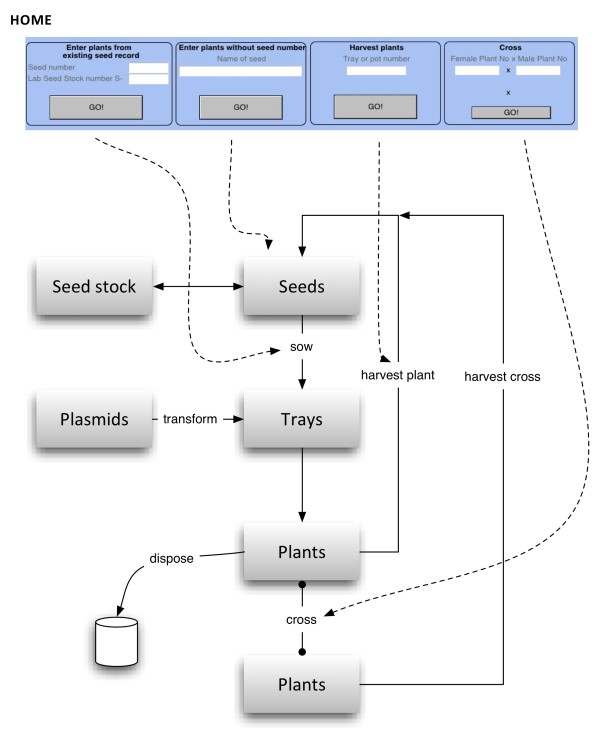
** Phytotracker workflow.** The Home screen is shown on the top and dashed arrows indicate which processes can be done through the Home screen. The logical work flow between Phytotracker tables is shown by straight arrows.

The ‘*Home’* screen of Phytotracker is the starting point displaying a selection of the most common tasks such as sowing, harvesting and crossing (Figure 2). For example, to sow seeds a user can simply enter the seed number if a seed batch already exists in the database and click GO to generate new plant records in the database. If information about a particular seed batch is not present in the database (for example when seeds are received from a stock centre), the user can enter a name for the seeds, in the ‘*Enter plants without seed number*’ box, which will then take the user to the *Seeds* table to submit more details. To harvest plants, the user enters the plant or tray ID number and the program will take the user to the harvesting screen where the user can harvest individual plants or a whole tray automatically generating and printing the corresponding seed labels, or simply indicate disposal of individual plants or the whole tray. To set up a cross between two plants, the ID numbers of the female and male plants can be entered which then will generate a seed record which corresponds to the seed pod or silique that will be harvested later.

### Seeds and Seed Stock tables

The *Seeds* table contains information about batches of seeds stored. Phytotracker allocates a unique number to each seed batch that is used throughout the system to identify plants originating from this seed batch. When seeds are harvested from a plant that is already in the system, information about the parent plant such as its name and ID number is stored in the seed record. A special type of seed record is a cross between two different parents, and in this case the information of both parents are stored. The ‘show ancestors’ button in the *Seeds* table will perform a search to find all the (female) antecedents of the seed record being browsed. This makes it easier to track the origin of a seed batch. If a seed batch is particularly useful for the lab, the user can copy the information into the *Seed Stock* table. This table is a valuable tool, representing that subgroup of seed lines of particular importance in a research group, for example, it can contain homozygous mutant combinations and transformants that are used by multiple researchers. The advantage is that the *Seed Stock* table can be browsed and searched more easily than the *Seeds* table, which, since it contains all seed batches ever harvested, makes it somewhat unwieldy for finding frequently used stocks. An additional advantage in having a *Seed Stock* is the ability to have a selection of curated seed batches. The *Seed Stock* table can contain more detailed information about the seed batches such as reference to journals, images and a field to record to which other research groups the seeds have been sent. It is important to note that a record in the *Seed Stock* is directly linked to the seed record such that the seeds will always keep their unique seed ID from the seed table in addition to the *Seed Stock*. This is necessary to keep track of the lineage of parent plants and their offspring.

### Pots and trays table

**Figure 3:**
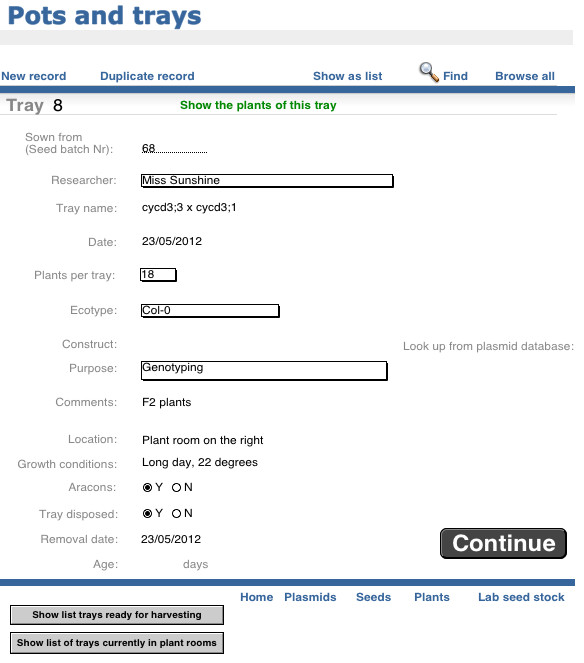
Pots and Trays screen.

The *Pots and Trays* table (referred to hereafter as *Trays* table) provides an easy platform for users to sow, keep track of and harvest several related plants at the same time, which would normally be physically associated in a single pot or tray in the growth facility. Its benefit is that it reduces data input if multiple plants are being grown, but is flexible with regard to the number of plants in a “tray” which can be set to one (as in a single pot-grown plant) or any other value. For example a user can sow a group of several plants from a single seed batch to bulk up seed stocks, use for crossing or to examine a segregating F2 population. When a user sows seeds a new tray record is automatically generated (Figure 3). Each tray record has a unique ID number and contains information about all individual plants in the “tray”. This way, a user does not need to enter information about each individual plant as Phytotracker uses the information from the tray table to enter the data for each individual plant. Key information about each plant is inserted from the *Seeds* table, but users can overwrite this and change or add more information such as the purpose, location and which construct was used in a transformation. Much of this information can be entered through customisable drop-down menus making the process quicker. This table also allows users to review the trays that are currently in the plant room and those that are ready for harvesting. This feature is particularly useful for technical staff responsible for the plant rooms or glasshouse. When the planting information is completed, the user simply clicks CONTINUE and the data is transferred to the *Plant* table which contains the information for each individual plant. Note that although the description refers to sowing seed, the process and screens are equally applicable to plants transferred into the growth facility from other sources, such as in vitro culture. The “growth conditions” field can be used to record specific information on growth medium, temperature, watering regimes etc., if required.

### Plant table

**Figure 4:**
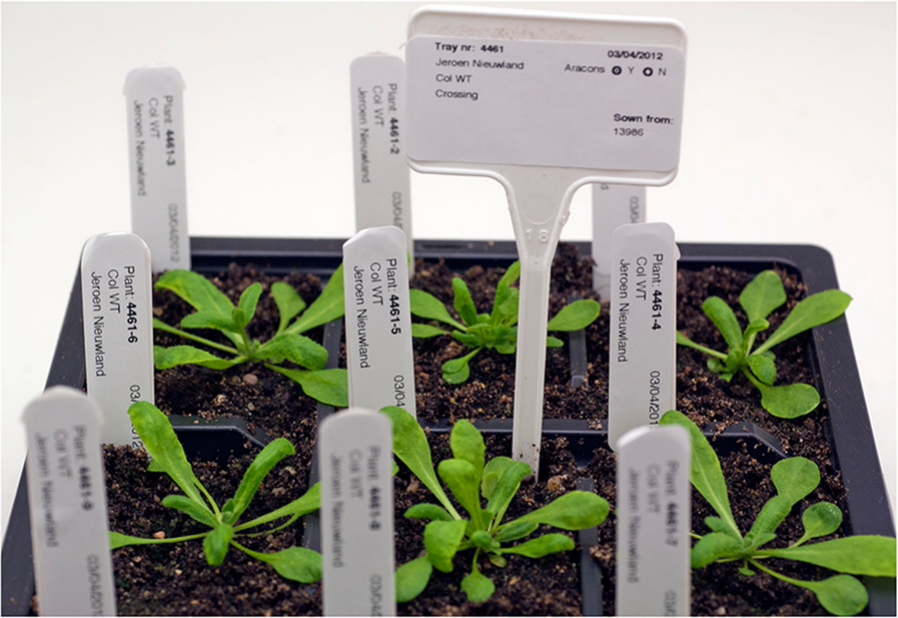
Example of a tray of 9 Arabidopsis thaliana plants showing the tray label and 9 individual plant labels.

Phytotracker allocates a unique ID for each plant in the plant table. This is derived from the tray number and the number of plants within that tray, so for example tray 4461 has 18 plants so plant 4461–5 is the 5^th^ plant in tray 4461 (Figure 4). These records display the information about that plant and link together all of the relevant information from the seeds table and tray table such as the age of the plant, researcher and from which seed batch it has been sown. From this screen the user can print a label for the tray, or if desired labels for each individual plant (Figure 4). At this point the user is ready to put the tray in the growth facility, each tray and plant being identified with a label. When the plants are fully grown, the user goes to the *Home* screen and enters the tray number to harvest the seeds of the plants. The “Samples prepared” field can be used to describe samples, such as RNA preparations made from specific plants.

### Harvesting seeds

**Figure 5:**
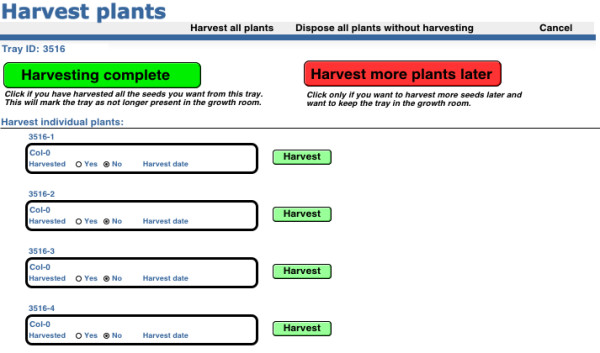
Harvest screen.

From the Home screen, the ‘Harvest plants’ button allows users to harvest their plants by entering the appropriate tray number, and the user will then arrive at the screen to harvest plants (Figure 5). The user has four options- to harvest the seeds from all the plants in the tray at once, to harvest some plants only and dispose the rest of the plants without harvesting, to harvest some plants only and put the tray back in the growth facility to harvest the rest later or finally to dispose all the plants in the tray without harvesting any seeds. If a user decides to harvest all the plants, he can click the top left ‘Harvest all plants’ button and the system will generate new seed records, print seed labels, and mark the plants as harvested in the *Plants* table and the tray as removed from the growth facility (disposed) in the *Tray* table. If no seed is to be harvested from any of the plants, the user clicks the top right ‘Dispose of all plants without harvesting’ button and the tray will be marked as disposed and no seed records will be generated. If only a selection of plants is harvested, the user clicks the green button next to the individual plant record(s) and seed records are generated appropriately with a corresponding seed label printed. When finished, the user can choose to keep the tray in the plant room, using the red ‘Harvest more plants later’ button, or mark the remainder of the plants in the tray as disposed using the green ‘Harvesting complete’ button. Either of these options exits the user from the harvesting screen.

### Plasmids table

**Figure 6:**
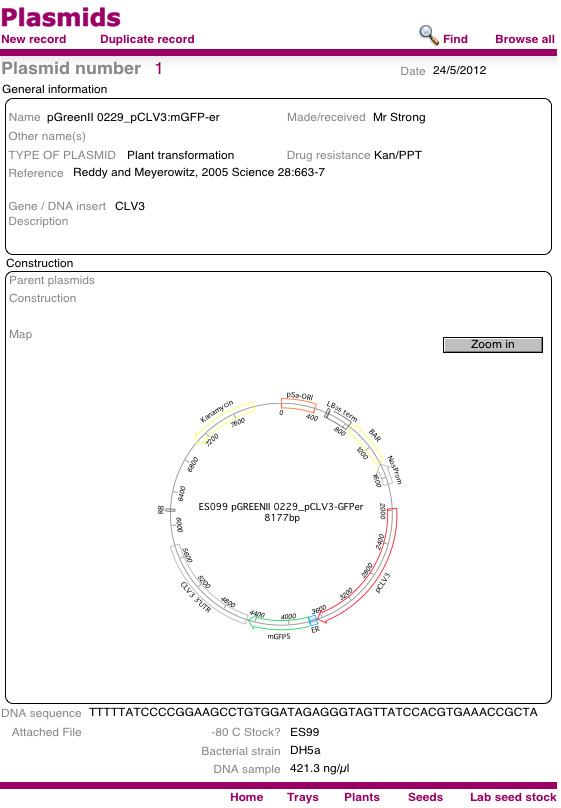
Plasmid screen.

The *Plasmids* table is not required for the Phytotracker system to function, but it allows users to link information about plasmids to their transformed plants. Users can store plasmid information such as name, resistance and a general description of the plasmid and how it was constructed. Furthermore it contains fields to include a picture of a map, sequence and information about strains and storage, or link to other files as desired (Figure 6).

### Record changes

Filemaker has a built-in user and password management system which allows to assign specific privileges to users, for example whether they are permitted to delete or modify records. In our research group, only the system administrator is allowed to delete records, but users are allowed to modify. Every change in a record is logged and can be accessed in the ‘Change log’ layout in each table.

### Online forum

Our aim is to provide a helpful tool to the plant community, which can be adapted to the specific needs of plant laboratories. Phytotracker is available through sourceforge.net, which is the leading open source software repository. On the dedicated project page, users have various options including a discussion forum and a possibility to upload their own version of Phytotracker.

### Future developments

As any live software product, Phytotracker will be subject to ongoing improvement. We plan to add more features such as a sample database linked to the plant and seed database, a better way to explore the progeny and parental lines from a particular plant or seed batch, barcode labelling, and a version of Phytotracker for mobile devices. The online forum will be helpful to interact with users and discuss further developments.

## Conclusions

Phytotracker was designed for facilitating the task of keeping record of growing plants, seed stocks and plasmids in molecular genetics labs. Ease of use and minimal user input were primary considerations during the design of the system. It is known that users should be involved in the development of a software product [8] and indeed our group has used and refined the system for over five years and it has helped us to manage thousands plants and seeds generated by many different users. The empty database can be downloaded from the link provided, along with a populated example. This site also provides a forum for user comments or improvements.

### Availability and requirements

Project name: Phytotracker

Project home page: http://sourceforge.net/projects/Phytotracker

Operating system(s): Windows XP or higher, Mac OSX 10.5 or higher

Other requirements: Filemaker Pro 8 or higher for standalone computer or for networked systems FileMaker Pro Advanced 8 or higher or FileMaker Server 8 or higher. FileMaker Pro 12 requires OSX 10.6. Dymo Labelwriter 450 or 450 Twin Turbo printers were used with Dymo 99017 for plant or seed labels and Dymo 11352 for tray labels.

License: GNU GPL

Any restrictions to use by non-academics: none

## Competing interests

The authors declare that they have no competing interests.

## Authors’ contributions

JN designed and programmed the database and drafted the manuscript. ES, AM and BdG helped in improving the software and drafted the manuscript. JAHM designed the database and drafted the manuscript. All authors read and approved the final manuscript.

## Supplementary Material

Additional file 1 ** Files, deposited at **http://sourceforge.net/projects/Phytotracker**.** Click here for file
