# Supplementary material for: Phytotracker, an information management system for easy recording and tracking of plants, seeds and plasmids
Source: Plant Methods. 2012 Oct 13;8:43. doi: 10.1186/1746-4811-8-43 (PMC3492177; doi:10.1186/1746-4811-8-43)
Supplement: Additional file 1 — Files, deposited at http://sourceforge.net/projects/Phytotracker. [file 1746-4811-8-43-S1.docx]

***Technical summary***

In this document we have compiled some technical background on Phytotracker and its components. The different layouts are described and the most important scripts. Some scripts are present in Phytotracker that are not currently used.

*Some notes for users*

There is a plethora of information available online on how to use Filemaker Pro in general and how to change and adapt a database. A good starting point is the official help page:

<http://www.filemaker.com/help/index.html>

The project home page on

https://sourceforge.net/projects/phytotracker/

contains a forum where users can discuss any issues. If required the corresponding author can be contacted as well. Always check the project page for most recent information and version of this document.

If a user decides to change Phytotracker, please make a copy of your database so no data will be lost.

To see the formal database and table relationships the user can go to File -> Manage -> database and then click ‘Relationships’

***Components***

**General scripts**

Go to X - to go various parts in Phytotracker, triggered by clicking the navigation buttons.

**Home**

*Layouts*

Home - Starting screen to access most parts of Phytotracker

My Plants - Summary of plants and seeds for individual researchers

*Scripts*

Go to X - to go various parts in Phytotracker, triggered by clicking the navigation buttons.

Look up X –updates the content of field X, by looking up related in table

Cross – starting the cross procedure, triggered by the GO button in the ‘cross’ panel, continues in the ‘seeds’ table with the script ‘paste female’

Continue cross – continue the cross procedure, handled over from ‘paste female’ in ‘Seeds’, continues in ‘paste male and continue’ in ‘seeds’.

Sow seed with seed number – triggered by GO button in ‘Enter plants from existing seed record’ panel. Will make new tray record using the script ‘sow seed with seed number’ in ‘trays’

Harvest plants – will start harvesting plants, triggered from the ‘Harvest plants’ panel. Continues in ‘Start harvest tray’ in the ‘Plants’ table.

Sow seed without seed number – triggered by GO button in ‘Enter plants without seed number’ panel. Will make new seed record using the script ‘Enter new seed’ in ‘seeds’ table.

Resize window – script used by layout to make the Home screen fit in window

Show home – shows home layout, used in the My plants layout

FamilyTree – under construction

**Plants**

*Layouts*

Plant list – default layout, showing plants in a list

Print – layout used in printing plant labels. Should be changed to adapt for printers different than described in manuscript.

Layout 5 – layout used to harvest plants (called by script)

Change log – shows the log of changes in the ‘Plants’ table

*Scripts*

Make plant tray – creates new plant records with names used in the ‘tray’ table. Script called by ‘ Make plants’ if user chooses to fill in the name for each plant.

Make tray no plant name – same as above but triggered if user does not chooses to fill in the name for each plant.

Show plants for tray – performs find to only show plants from a specified tray, called by various other scripts

Harvest plants – harvests all plants, triggered by clicking ‘Harvest all plants’ in the harvesting layout (Layout 5).

Dispose plants – disposes plants without harvesting seeds (and creating new seed records. Triggered by ‘Dispose all plants without harvesting’ in harvesting layout.

Harvesting complete – marks the tray as removed by calling ‘Dispose tray’ in ‘Tray’ table. Triggered by clicking ‘Harvesting complete’.

Harvest more plants later – goes back to plant list layout, used to exist Harvesting screen without marking trays as removed. Triggered by clicking ‘Harvest more plants later’.

Print plant labels current view – prints plant labels of the plants currently in viewed

Perform find for duplicates – checks if plants of a certain tray are already in database. Used in script ‘Make plants’ from ‘Trays’.

Start Harvest Tray from plant database - triggered by clicking ‘Harvest plants in this tray’. Performs find of current tray and goes to Layout 5.

Harvest this plant – creates new seed record with information from plant record. Triggered by clicking ‘Harvest’ in Layout 5.

Print tray label – will call the script in ‘Tray’ table to print the tray label.

**Pots and trays**

*Layouts*

Screen – main screen showing tray and pot information

Label - layout used in printing tray labels. Should be changed to adapt for printers different than described in manuscript

List trays – shows trays in a list

Change log – shows the log of changes in the ‘Trays and pots’ table

*Scripts*

Print label – prints a tray label

Make plants – After filling in details about the trays, this script will make individual plant records in the ‘plant’ table. It will take information from how many plants per tray and will ask if the tray name should be used for each plant record.

Show this tray in plant database – performs a find in the ‘plants’ table and shows only the plants from the current tray

Count X – performs find using researcher’s name and if trays are active, harvested or all and returns number of found records

List old trays – go to ‘List trays’ layout and sorts on age showing oldest trays first

List current trays - go to ‘List trays’ layout and shows only trays that are not marked as removed

Sow seed with seed number - starts new record using a seed number and take information from the seed table to fill the ‘plants and pots’ table with information. Used from the ‘Home’ table.

**Seeds**

*Layouts*

Layout 1 - main screen with seed information

Print label - layout used in printing seed labels. Should be changed to adapt for printers different than described in manuscript

Crossed plant – can only be seen if male and female parents are not the same

Print label cross- layout used in printing cross labels. Should be changed to adapt for printers different than described in manuscript

Ancestors – used to show the ancestors of a particular seed batch, used in the relevant script

Tree – under construction

Change log – shows the log of changes in the ‘Trays and pots’ table

*Scripts*

Make new record and enter plant detail – used from Home table to start a new seed record

Print label – prints a seed label

Print label cross – prints a cross label

Transfer to Lab stock – used if user clicks ‘submit as Lab Stock’. Information is copied to a new Lab Seed Stock record

Paste female – see the ‘cross’ script in ‘Home’ table

Paste male and continue – see the ‘cross’ script in ‘Home’ table

Check cross – is performed every time to choose between the Layout 1 layout and ‘Crossed plant’ layout. Checks if male and female parents are the same

Ancestors – performs a looped find to display the ancestors of the current seed batch being viewed

**Plasmids**

*Layouts*

Layout 1 – main screen

Full screen picture – displays the map full screen

Full sequence – shows the full sequence

Change log – shows the log of changes in the ‘plasmids’ table

**Lab seed stock**

*Layouts*

Layout 1 – main screen

Print labels - layout used in printing seed stock labels. Should be changed to adapt for printers different than described in manuscript

Change log – shows the log of changes in the ‘lab seed stock’ table
